# Decoding Chinese speech across multiple neural conditions via EEG: dataset construction and interpretability driven spatial optimization

**DOI:** 10.3389/fpsyg.2026.1833448

**Published:** 2026-07-27

**Authors:** Haoming Wang, Gaoyuan Zhang, Xurong Xie, Ying Qin, Tian Zheng, Chao Zhou, Jin Huang, Feng Tian, Shikui Wei

**Affiliations:** 1 Institute of Information Science, Beijing Jiaotong University, Beijing, China; 2 Visual Intelligence +X International Cooperation Joint Laboratory of MOE, Beijing, China; 3 Institute of Software, Chinese Academy of Sciences, Beijing, China

**Keywords:** brain computer interface, Chinese mandarin, EEG, model interpretability, multi-task learning, speech decoding

## Abstract

The integration of artificial intelligence (AI) and brain-computer interfaces (BCIs) technologies shows great potential in assisting patients with speech impairments and improving cognitive-linguistic decline. Electroencephalogram (EEG) based BCIs, characterized by non-invasiveness, low cost, and high temporal resolution, hold significant application value in speech decoding and cognitive rehabilitation. Currently, most mainstream public EEG datasets rely on Western languages. As a tonal language, Chinese Mandarin differs significantly from Western languages in speech production mechanisms, making existing data insufficient to support future BCI research for Mandarin-speaking patients. To address this gap, we establish a systematic Mandarin EEG dataset and conduct effective speech decoding and related analyses. We design four distinct experimental conditions, namely overt, overt-noisy, intend, and imagine, to simulate different types of speech disorders in clinical scenarios. Using typical Mandarin tonal-vowels and common vocabularies as stimuli, we construct an EEG dataset collected from a healthy adult. We evaluate the speech decoding performance using short-time Fourier transform combined with support vector machine (STFT-SVM) and EEG-Conformer models. Furthermore, we design a multi-task architecture based on the EEG-Conformer to perform a unified decoding task for the two stimulus types and a classification task across the four dataset conditions. To interpret the model, we combine Shapley value computation and decision trees to calculate the importance of different electrodes during classification. Experimental results show that the models achieve effective decoding on our dataset. The EEG-Conformer model performs significantly above chance level across all data, reaching an accuracy of 69.83% in normal speaking conditions and up to 61.46% in conditions simulating speech disorders. In the multi-task setting, the classification accuracy across different conditions exceeds 97%. By utilizing the important electrodes identified through interpretability methods as new feature inputs, the classification performance further improves even with a reduction of over 50% in the channels. These results demonstrate the potential of neural signal decoding technologies in communication assistance, reveal the decodability of Chinese Mandarin EEG datasets, and provide feasible recommendations for the future design of Chinese BCI applications.

## 1 Introduction

Language represents the primary medium for human social interaction, playing a critical role in both information transmission and emotional expression. The frontal lobe of the human brain, serving as the principal module for language processing, provides the neural computing capacity required for the comprehension and generation of language (Friederici, 2011). Clinical conditions such as stroke, traumatic brain injury, tumors, and amyotrophic lateral sclerosis (ALS) can cause damage to the motor control pathways of the brain, frequently resulting in severe speech impairments, particularly dysarthria (Duffy, 2012). These impairments significantly limit patients' daily communication and overall quality of life. However, the effective restoration of motor speech functions for individuals with profound speech impairments remains a complex and persistent challenge.

Brain-computer interface (BCI) technology establishes a direct communication pathway between the brain and external devices by interpreting electrophysiological signals from the central nervous system. By capturing neural signal fluctuations within the brain regions responsible for speech production and translating them into interpretable communication signals, BCI facilitates the restoration of communicative abilities in patients with speech disorders (Willett et al., 2023; Metzger et al., 2023). This approach is distinct from direct medical intervention, it functions as an assistive technology for the rehabilitation of speech impairments (Chaudhary et al., 2016).

Driven by the rapid progression of BCI technologies, a diverse array of neural modalities has been harnessed for speech decoding, including electroencephalography (EEG), functional magnetic resonance imaging (fMRI) (Logothetis, 2008), and electrocorticography (ECoG) (Dubey and Ray, 2019; Tang et al., 2023). Notably, Naselaris et al. (2011) elucidated the complementary relationship between encoding and decoding models within fMRI research. Their findings demonstrate that while both approaches can effectively interrogate neural representations, encoding models provide a more comprehensive functional characterization of brain regions, thereby establishing a rigorous modeling framework that transitions from voxel-wise encoding to stimulus decoding. Similarly, the work of Wang et al. (2011) confirms the feasibility of semantic category decoding from ECoG signals. By identifying robust high-gamma band activations in language-related cortical regions and analyzing their distinct spatiotemporal dynamics, they validated that machine learning classifiers can reliably predict semantic content from distributed cortical activity, providing a foundational empirical basis for ECoG-driven communication systems.

Despite the high spatial resolution and robust signal quality offered by fMRI and ECoG, their widespread clinical and deployment is frequently constrained by the necessity for invasive surgical procedures or restrictive, high-cost acquisition environments. Consequently, EEG has emerged as a highly compelling alternative due to its non-invasive nature, exceptional temporal resolution, and suitability for portable BCIs. In parallel, the field has witnessed a significant surge in EEG-based analysis, facilitated by sophisticated signal processing and deep learning architectures that allow for the nuanced decoding of internal cognitive states (Défossez et al., 2023; Duan et al., 2023). For instance, Wang and Ji (2022) addressed the challenges of open-vocabulary EEG-to-text decoding by implementing a sequence-to-sequence learning framework. By integrating EEG data recorded during natural reading with the pre-trained language model BART, they successfully decoded neural signals into coherent natural sentences and achieved a zero-shot ternary sentiment classification F1 score of 55.6%. However, it is imperative to recognize that these methodologies, particularly those leveraging pre-trained models like BART, are fundamentally grounded in English linguistic structures and Western datasets.

To overcome the constraints of dataset-specific models, the Large Brain Model (LaBraM) (Jiang et al., 2024) was developed to enhance scalability and generalizability across diverse neural tasks. By segmenting raw EEG signals into channel patches and utilizing vector-quantized neural spectrum prediction, LaBraM trains a neural tokenizer to convert patches into discrete neural codes, followed by pre-training via masked neural code prediction. While this model achieves superior performance in tasks such as emotion recognition and gait prediction, its pre-training regime remains primarily focused on non-tonal linguistic patterns, leaving the unique challenges of tonal languages largely unaddressed.

Currently, Chinese, as one of the most widely spoken languages globally, exhibits fundamental phonological distinctions from English (Liu and Fung, 2004), especially regarding its tonal nature. Nevertheless, most publicly available EEG datasets are predominantly derived from non-tonal languages (DaSalla et al., 2009; Nieto et al., 2022; Zhao and Rudzicz, 2015). This linguistic specificity poses significant challenges for the direct application of neural decoding models and feature extraction methods to Chinese, as these techniques were originally developed for non-tonal languages. Consequently, the progress of research in Chinese BCIs has been substantially hindered. While some public datasets incorporate Chinese speech, they primarily focus on sentence-level decoding and often neglect the role of vowels (Mou et al., 2024; Zhang et al., 2024). Vowels, such as /a/, /o/, /e/, /i/, /u/, and /ü/, serve as the core constituents of all syllables in Chinese Mandarin, acting as the structural foundation of speech production. More importantly, in a tonal language like Mandarin, these vowels act as the primary phonetic carriers for tones. The pitch variations inherent in these tones are not merely prosodic features but are semantically contrastive, meaning that a change in tone fundamentally alters the lexical meaning of a syllable. Therefore, investigating vowels in isolation is insufficient, it is the integrated decoding of tonal-vowels that provides a critical window into the neural representations underlying Chinese Mandarin.

Furthermore, various types of EEG datasets have been constructed for individuals with diverse speech disorders. For example, Nguyen et al. (2018) distinguished between three distinct imagined speech tasks: short words, vowel phonemes, and longer words. Similarly, ChineseEEG (Mou et al., 2024) established an overt speech EEG dataset based on sentences from novels for children for semantic alignment and neural decoding. Despite these advancements, more complex clinical conditions must be considered. Specifically, certain patients may attempt articulation while lacking auditory feedback, a condition that remains unaddressed in existing datasets. To address these gaps, we introduce a multi-condition EEG dataset for Chinese Mandarin speech, encompassing both tonal-vowels and vocabularies across four conditions: *Overt, Overt-noisy, Intend*, and *Imagine*. Our methodological framework employs two distinct classification approaches: STFT-SVM (Cortes and Vapnik, 1995) and a hybrid architecture called EEG-Conformer (Song et al., 2022), which combines convolutional layers with Transformers (Vaswani et al., 2017). Specifically, this study aims to address the following three research questions: (1) Whether the multi-condition EEG dataset design is valid and capable of achieving reliable decoding performance across mainstream architectures. (2) Whether the joint training of EEG data across different linguistic granularities (specifically vocabulary and tonal-vowel) can serve as a complementary data augmentation strategy to overcome current scale limitations and extract robust shared neural representations, thereby improving the overall decoding efficacy for Mandarin speech. (3) Whether model interpretability methods can accurately quantify electrode importance to guide critical site selection, thereby reducing the required number of electrodes to lower hardware costs and enhance the generalizability of BCI systems.

Our contributions are as follows:

• To the best of our knowledge, we were the first to develop multi-condition Chinese Mandarin EEG dataset and conduct systematic research under various conditions, applied it to the Chinese. We considered individuals who could speak normally but had no auditory feedback, and thus designed the experimental condition called overt-noisy.

• We conducted a comprehensive evaluation of the decoding performance on the dataset using STFT-SVM and EEG-Conformer frameworks to investigate the variations in EEG neural decoding efficacy across different conditions. When using the EEG-Conformer method, the accuracy of our dataset reached up to 69.83%.

• Based on EEG-Conformer, different multi-task structures were designed in different data analysis dimensions. We jointly trained the model on data from the same condition but different tasks, enabling it to simultaneously learn the neural features of tonal-vowels and vocabularies. This allowed us to investigate whether data from distinct tasks could mutually enhance model performance. Data of the same task but different conditions were jointly trained to verify the rationality of our multi-condition Chinese Mandarin dataset design. The best average accuracy of the multi-task model across different tasks reached 45.66%. The classification accuracy between different conditions exceeded 97%.

• For our dataset, we trained Random Forests(RF) (Breiman, 2001) and calculated the SHapley Additive exPlanations (SHAP) values (Lundberg and Lee, 2017), obtaining the importance rankings of the channels from different brain regions, which revealed the participation degrees of different brain regions in the process of Chinese language decoding. Furthermore, we used the crucial electrodes for the classification task and achieved excellent classification results.

## 2 Materials and methods

### 2.1 Participants

To minimize the confounding effects of inter-subject variability and neural manifold misalignment on the decoding performance, this study adopted a deep single-subject experimental design. Data were collected from a 22-year-old right-handed male participant. The subject possessed a native command of Chinese Mandarin, reported no history of neurological or psychiatric disorders, and passed an initial audiometric screening to ensure normal hearing and vision. Prior to the formal acquisition sessions, a pilot trial was conducted to verify the stability of EEG acquisition system and the subject's adherence to the experimental protocol. The participant provided written informed consent before the commencement of the study, which was conducted in accordance with the Declaration of Helsinki.

### 2.2 Experimental stimuli

The speech stimuli were categorized into two distinct groups: tonal-vowel and vocabulary. The tonal-vowel set was designed to capture the phonological complexity of Chinese Mandarin by combining 6 fundamental vowel phonemes with 4 tones, resulting in 24 unique tonal-vowel stimuli. Each stimulus was presented across 30 trials, yielding a total of 720 trials and approximately 7.28 hours of high-quality neural recordings. The vocabulary set consisted of 6 Chinese vocabularies selected based on their high frequency in activities of daily living and clinical hospital scenarios, such as “eating” and “medical dressing change.” Each vocabulary was repeated over 80 trials, totaling 480 trials and 4.7 hours of high-quality neural recordings. The comprehensive list of stimuli is detailed in Tables 1, 2.

**Table 1:** Comprehensive list of Mandarin tonal-vowel stimuli.

Vowel	Tone 1	Tone 2	Tone 3	Tone 4
a	ā	á	ǎ	à
o	ō	ó	ǒ	ò
e	ē	é	ě	è
i	ı̄	í	ǐ	ì
u	ū	ú	ǔ	ù
ü	ǖ	ǘ	ü^	ǜ

**Table 2:** Comprehensive list of Chinese vocabulary stimuli.

Category	Stimuli
Daily life	吃饭 (Have meal), 喝水(Drink), 睡觉 (Sleep)
Clinical setting	厕所(Toilet), 头疼(Headache), 换药 (Change dressing)

### 2.3 Multi-condition experimental design

To simulate the diverse clinical manifestations of speech communication disorders, we implemented a multi-condition framework comprising four distinct states. The distribution of valid recording hours across these conditions is summarized in Table 3.

**Table 3:** Distribution of valid recording hours.

Dataset structure	Healthy conditions	Simulated dysarthria conditions
Tonal-vowel	Overt(1.64h)	Overt-noisy(2.1h), Intend(1.87h), Imagine(1.67h)
Vocabulary	Overt(1.15h)	Overt-noisy(1.15h), Intend(1.17h), Imagine(1.23h)

• Overt: This condition required the participant to produce speech naturally and clearly, serving as the physiological ground truth for neural activity during successful articulation.

• Overt-noisy: This condition is designed to simulate the processing of auditory feedback that is impaired or distorted, which is frequently observed in specific disorders of speech, such as conduction aphasia (Ardila, 2010). Under this condition, the participant performed overt speech while wearing headphones that delivered calibrated white noise. The primary objective of introducing such noise was to actively interfere with and mask the normal auditory feedback of the participant. Rather than simulating a state of complete auditory deprivation, the continuous noise distorts the input of perceived speech, thereby creating a mismatch in sensory perception. This condition compels the brain to process auditory information that is blurred or degraded during the production of speech, which mimics the compromised integration between the auditory and motor systems experienced by patients with impairments of speech.

• Intend: This condition required the participant to attempt articulation by engaging laryngeal muscles without producing successful phonation, thereby simulating patients with severe dysarthria who retain motor intent but fail in execution.

• Imagine: This condition involved purely covert speech, where the participant mentally rehearsed the stimuli without any muscular movement, simulating the complete loss of motor pathways typical of late stage amyotrophic lateral sclerosis.

### 2.4 Experimental paradigm and acquisition

**Figure 1:**
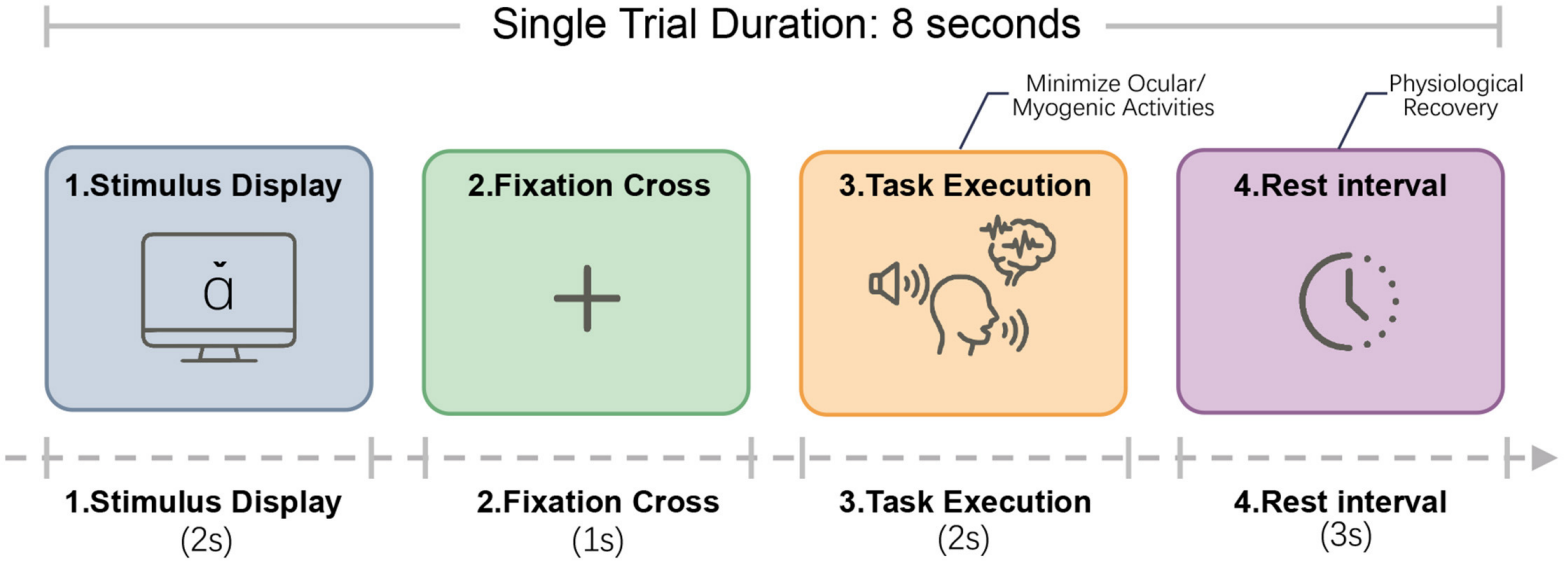
The total duration of each trial is 8 s, divided into four distinct phases: (1) Stimulus Display (2 s): presentation of the speech target; (2) Fixation Cross (1 s): a period to stabilize the participant's gaze and minimize anticipatory artifacts; (3) Task Execution (2 s): the active window where the participant performs the required speech task while instructed to minimize ocular and myogenic activities; (4) Rest Interval (3 s): a period designated for physiological recovery and mental reset before the subsequent trial.

**Figure 2:**
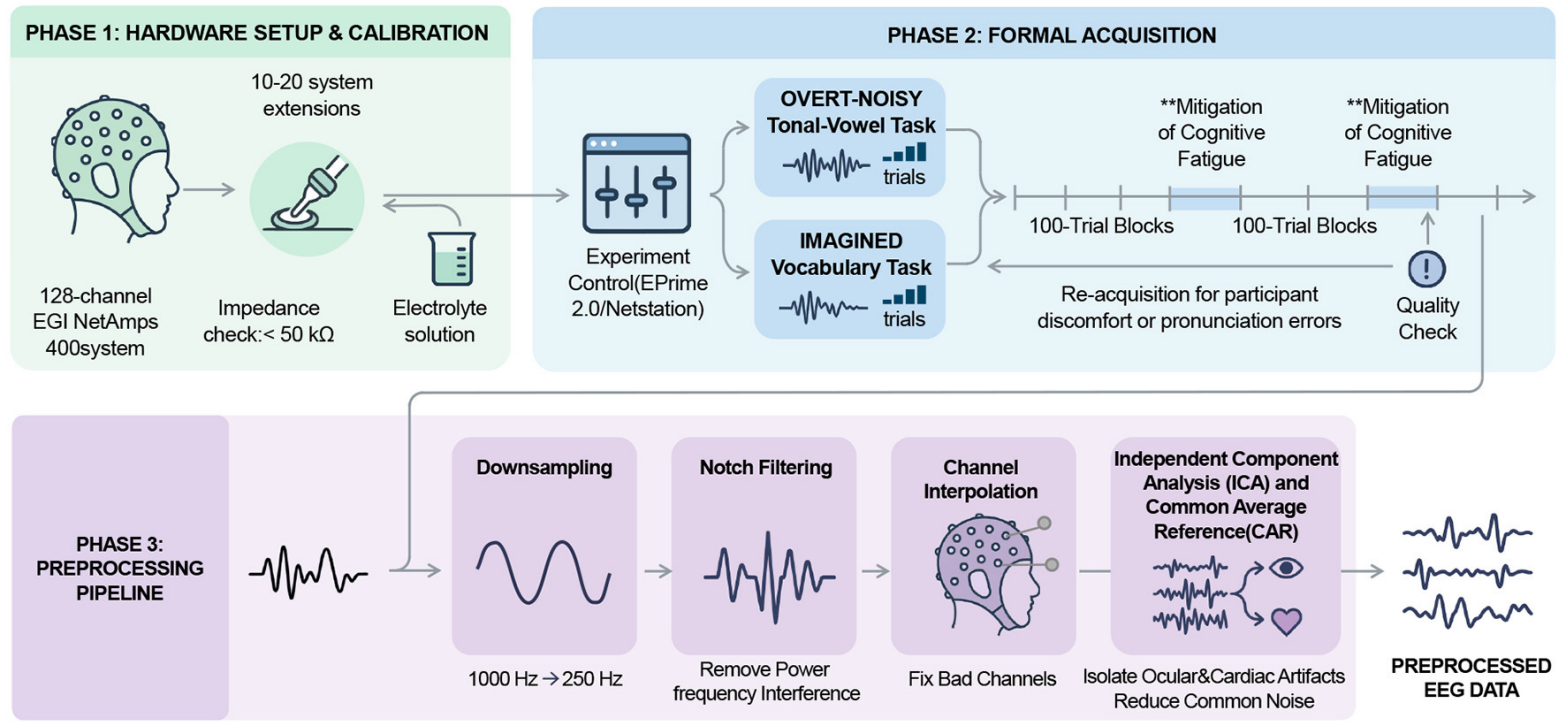
The experimental workflow is organized into three phases: **Phase 1** (Hardware setup & calibration): utilization of a 128-channel EGI system with electrode impedance maintained below 50 kΩ. **Phase 2** (Formal acquisition): execution of tonal-vowel and vocabulary tasks controlled by E-Prime 2.0, structured in 100-trial blocks with rest periods to mitigate cognitive fatigue and quality checks to ensure data integrity. **Phase 3** (Preprocessing pipeline): sequential signal processing steps including downsampling (from 1,000 Hz to 250 Hz), notch filtering for powerline interference removal, channel interpolation for bad channels, and ICA was used to isolate and remove ocular/cardiac artifacts, while CAR was applied to reduce common noise and improve signal quality.

The experimental paradigm followed the BCI Competition IV (Tangermann et al., 2012) framework to ensure temporal precision and cognitive load balance. Each trial initiated with a stimulus display, which lasted 2 s, followed by a fixation cross of 1 s to stabilize the participant's attention and minimize anticipatory artifacts. During the subsequent task execution window of 2 s, the participant performed the required speech task under one of the four specified conditions. An interval of 3 s between trials was provided for physiological recovery and mental reset. Throughout the task, participants were instructed to maintain a neutral gaze and minimize ocular and myogenic activities. Figure 1 illustrates the process of one trial within the experimental paradigm. Figure 2 presents the overall flowchart of data acquisition and preprocessing.

Neural data were acquired using a high-density 128-channel EGI NetAmps 400 system. Electrodes were positioned according to the international 10–20 system extensions, with impedances maintained below 50 kΩ to ensure signal integrity. The paradigm was controlled via E-Prime 2.0, with real-time synchronization achieved through Netstation software. The process was partitioned into three phases. Phase 1 involved hardware calibration and impedance adjustment using a specialized electrolyte solution. Phase 2 comprised the formal acquisition, where sessions were interrupted every 100 trials to mitigate the effects of cognitive fatigue. Notably, datasets for the Overt-noisy tonal-vowel task and Imagined vocabulary task were re-acquired following participant-reported discomfort or pronunciation errors to ensure data consistency. Phase 3 focused on preprocessing, where the initial signal of 1,000 Hz was downsampled to 250 Hz to optimize computational efficiency. The pipeline further included notch filtering to eliminate interference from the power line, the interpolation of bad channels, and Independent Component Analysis (ICA), which was employed to isolate and remove artifacts originating from ocular and cardiac activities. Finally, the Common Average Reference (CAR) is applied for re-referencing the entire brain to eliminate common noise. The detailed preprocessing steps can be found in Chapter 3.

## 3 Neural decoding framework

### 3.1 Overview of the multi-condition speech decoding

This section presents the computational architecture developed for the multi-condition decoding of Chinese Mandarin speech. Building upon the dataset established in previous section, the current framework integrates traditional linear classification methods with deep learning paradigms. The objective is to leverage shared neural representations across diverse conditions representing potential clinical states, which enables effective neural decoding of Chinese Mandarin speech.

**Figure 3:**
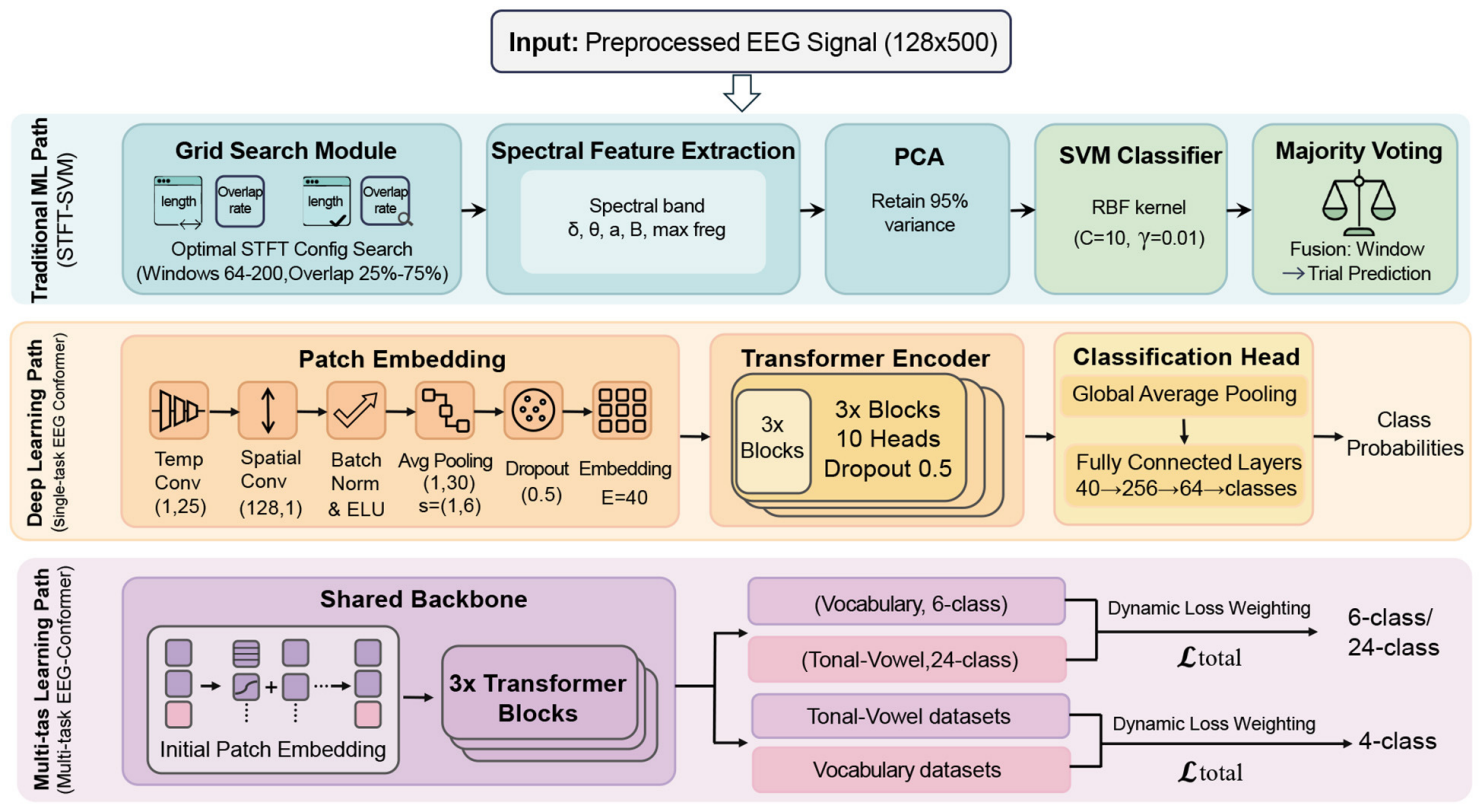
The framework processes preprocessed EEG signals of dimension 128 × 500 through three specialized paths: (1) Traditional ML Path (STFT-SVM): This pipeline utilizes a grid search module (window lengths 64–200, overlaps 25%–75%) to identify the optimal STFT configurations. Following spectral feature extraction across multiple frequency bands, PCA is applied to retain 95% of the variance. Classification is performed by a SVM with an RBF kernel (*C* = 10, γ = 0.01), using majority voting for trial-level prediction. (2) Deep learning path (single-task EEG-conformer): This path extracts deep neural representations starting with Patch Embedding, which includes temporal and spatial convolutions, batch normalization, and average pooling. The features are then processed by a Transformer Encoder consisting of 3 blocks and 10 attention heads with a 0.5 dropout rate. The Classification Head employs global average pooling and fully connected layers (40 → 256 → 64 → classes) to generate class probabilities. (3) Multi-task learning path (multi-task EEG-conformer): built upon EEG-conformer, this architecture incorporates an adaptive parameter-sharing strategy with task-specific branching. It jointly models vocabulary (6-class) and tonal-vowel (24-class) data, or identifies the specific experimental condition (4-class) from aggregated datasets. The framework is optimized via a joint loss function (L_*total*_) with Dynamic Loss Weighting to balance the learning objectives of different tasks.

The input to the decoding pipeline consists of preprocessed high-density EEG tensors, denoted as a series of *X*∈**R**^*C*×*T*^, where C = 128 and T = 500 representing the spatial manifold of the electrode channels and T signifies the temporal samples within the 2 s task window. These neural signals encapsulate two primary linguistic categories: a set comprising 24 tonal-vowel combinations and a set consisting of 6 daily-life vocabularies. Each category is recorded under four simulated clinical conditions: *Overt, Overt-noisy, Intend*, and *Imagine*. The framework employs a multi-class SVM with a radial basis function kernel (Cortes and Vapnik, 1995). To optimize time-frequency resolution, a grid search is conducted over various combinations of sliding window lengths and step sizes within the Short-Time Fourier Transform (STFT) to identify the optimal parameter configuration (Wang et al., 2019). Simultaneously, an EEG-Conformer network (Song et al., 2022) is constructed and trained to extract deep neural representations from the raw signals. The schematic diagram of the entire architecture is shown in Figure 3. To address the inherent complexity of these neural manifolds, our framework is applied to three different types of decoding tasks.

• Tonal-Vowel task: Focusing on vowels, the core constituents of all syllables in Chinese Mandarin, a series of single-task experiments is conducted. The evaluation is systematically decomposed into vowel recognition across six categories, tone recognition across four categories, and tonal-vowel decoding across twenty-four categories. This task design aims to investigate the dataset from multiple dimensions and explore the underlying representations encoded within neural signals during speech processing.

• Vocabulary task: This task focuses on decoding six specific Mandarin vocabularies, selected based on their relevance to daily life and clinical environments. This experimental design aims to establish a practical context for BCI applications intended for Mandarin speakers with speech impairments.

• Multi-Task learning: Building upon the EEG-Conformer architecture, two multi-task learning paradigms are introduced. The first paradigm jointly models vocabulary and tonal-vowel data under a single experimental condition. Unlike traditional approaches that rely on fixed global sharing, this paradigm incorporates an adaptive parameter-sharing strategy that allows for task-specific branching at various architectural depths, with the entire framework being optimized through a joint loss function. The second paradigm aggregates data from either the vocabulary task or the tonal-vowel task across all four experimental conditions. This configuration formulates a four-class classification problem to identify the specific condition (*Overt, Overt-noisy, Intend*, and *Imagine*) from the aggregated data.

### 3.2 Model architecture and experimental settings

#### 3.2.1 STFT-SVM architecture

**Preprocessing**: A unified preprocessing pipeline is established for the input data of both STFT-SVM and EEG-Conformer. Initially, EEG channels are isolated from the raw recordings, and the reference electrode VREF is removed. A notch filter at 50 Hz is applied to eliminate power line interference, while a bandpass filter constrains the signal to a frequency range of 0.1–30 Hz. To maintain data integrity, bad channel interpolation is performed, where missing electrode signals are reconstructed using a fit derived from adjacent channels. Following the evaluation of various downsampling rates, a frequency of 250 Hz demonstrates optimal classification accuracy across all conditions. The final stages involve ICA and re-referencing. Since scalp EEG signals are a linear superposition of diverse neural activities and physiological noise, ICA is utilized to identify and remove independent components associated with ocular, myogenic, and cardiac artifacts. Furthermore, to address the spatial bias and localized noise introduced by the physical Cz reference electrode used during acquisition, the data are re-referenced by the CAR. This procedure utilizes the instantaneous mean voltage across all electrodes as a virtual reference, effectively suppressing global noise.

Following preprocessing, a feature extraction framework based on the STFT captures the temporal dynamics of the EEG signals. Given the distinct neural oscillations across different frequency bands during speech tasks, spectral power features are extracted from four primary bands to reduce complex information into physiologically meaningful representations: δ (1–4 Hz), θ (4–8 Hz), α (8–12 Hz), and β (12–30 Hz) (Buzsáki, 2006). The dominant frequency within the 1–30 Hz range is also included to characterize peak oscillatory activity, which improves class separability.

Since the effectiveness of STFT-based decoding is highly sensitive to the selection of window length and overlap ratio, a grid search identifies the optimal parameters for the Mandarin speech decoding task. The parameter space for this search includes window lengths ranging from 64 to 200 samples and overlap ratios from 25% to 75% (Wang et al., 2019). For each trial, the signal is segmented into sliding windows, and features from all 128 channels are concatenated into a high-dimensional vector. To balance energy magnitudes across frequency bands and enhance model generalization, a processing pipeline is established where features are first standardized via a standard scaler and subsequently processed using Principal Component Analysis (PCA) to retain 95% of the cumulative variance. Following the stage of dimensionality reduction, the SVM with a Radial Basis Function (RBF) kernel is employed as the classifier. This selection is justified by the capacity of the RBF kernel to project the resulting spectral features into a space characterized by nonlinearity, thereby enabling the model to capture non-linear decision boundaries that persist despite the dimensionality reduction process. During training, each constituent window {*w*_*i*, 1_, *w*_*i*, 2_, …, *w*_*i, N*_} is treated as an independent sample in the feature space. This strategy serves as a form of temporal data augmentation that effectively expands the available sample size by a factor of N. Because EEG signals exhibit a strong lack of stationarity, neural firing patterns are not constant but fluctuate even when a subject maintains focus on a specific task. Multi-window training enables the STFT-SVM to learn these diverse localized states within a trial. To ensure evaluation accuracy, a 5-fold Group cross-validation scheme is implemented. The groups are defined at the trial level to ensure that all augmented windows from the same trial are strictly partitioned into either the training or testing set within any given fold, thereby preventing data leakage. In this configuration, 80% of the augmented windows are used for training and 20% for evaluation. The specific configuration is shown in Table 4.

**Table 4:** Hyperparameters and architectural configurations for STFT-SVM.

Component	Parameter	Value/configuration
Preprocessing	Sampling rate *f*_*s*_	250 Hz
	Band-pass filter	0.1–30 Hz
	ICA	45 components
	Reference	Common average reference
Feature extraction	Window length *L*	Grid search: {64, 100, 128, 150, 200}
	Overlap ratio	Grid search: {25%, 50%, 75%}
	Analysis window	0–2.0 s (0–500 samples)
	Frequency bands	δ, θ, α, β and peak frequency
	Dimensionality reduction	PCA (95% variance retained)
Classifier	Kernel function	RBF
	Regularization *C*	10
	Kernel coefficient γ	0.01
	Decision mechanism	Window-level majority voting

In the inference phase, taking the vocabulary classification task as a representative case, the classifier generates a predicted label ŷ_*i, j*_ for each window *j* of a test trial *T*_*i*_. These individual predictions are then aggregated through a majority voting process to produce the final trial-level classification Ŷ_*i*_:


$$\begin{array}{l} {Ŷ}_{i}=\underset{k\in \left\{1,\ldots ,6\right\}}{\text{argmax}}\overset{N}{\underset{j=1}{\sum }}I\left({ŷ}_{i,j}=k\right) \end{array}$$ (1)

where *I*(·) denotes the indicator function. This two stage approach, training on augmented windows and evaluating via a consensus at the trial level, combines the benefits of a large, diverse training set with a robust decision process that is resistant to noise, thereby improving the overall reliability of the neural decoding system.

#### 3.2.2 EEG-conformer architecture

The architecture of EEG-Conformer is employed to further explore the spatiotemporal representations of Mandarin speech tasks. The input data follow the same preprocessing steps as described in the previous section. To ensure numerical stability and enhance model convergence, the data are standardized such that each trial has a zero mean and unit variance before being fed into the network.

The initial stage of the model consists of a patch embedding module designed to extract shallow features. This module utilizes a temporal convolution layer with a kernel size of (1,25) to capture local dynamics, followed by a spatial convolution layer with a kernel size of (128,1) across all C channels to aggregate information from the entire scalp. Subsequent to these convolutions, batch normalization and the exponential linear unit (Clevert et al., 2015) activation function are applied. An average pooling layer with a kernel size of (1,30) and a stride of (1,6) is then used to reduce the temporal dimension, followed by a dropout layer with a rate of 0.5 to mitigate overfitting. The resulting feature maps are projected into an embedding space of dimension E, where E = 40 in the primary configuration.

The core of the architecture is a Transformer encoder comprising a series of identical blocks. Each block integrates a multi-head self-attention (MSA) mechanism and a feed-forward network (FFN). The MSA allows the model to capture long-range temporal dependencies by computing the relationships between different time steps in the embedding sequence. Specifically, queries, keys, and values are linearly projected from the input, and attention scores are calculated using a scaled dot-product. Layer normalization and residual connections are implemented around both the MSA and FFN components to facilitate gradient flow and stabilize the training process. The encoder consists of D = 3 blocks, each utilizing 10 attention heads and a dropout probability of 0.5.

The final classification head utilizes a global average pooling layer to collapse the temporal dimension into a fixed-length vector. This vector is then processed by a multi-layer perceptron (MLP) consisting of two hidden layers with 256 and 64 units, respectively, employing ELU activations and dropout for regularization. The output layer produces the final class probabilities. The model is trained using the cross-entropy loss and the Adam optimizer with a learning rate of 0.0002 and momentum coefficients β_1_ = 0.5 and β_2_ = 0.999. Training was performed for a maximum of 500 epochs, incorporating an early-stopping mechanism based on the accuracy of the validation set. The state of the model was saved whenever the validation accuracy improved relative to the previous best performance. This strategy effectively mitigates overfitting by selecting the model with the best validation performance rather than at the final training epoch. The specific configuration is shown in Table 5. All models utilize a shared architecture comprising the patch embedding, the transformer encoder, and identical optimization configurations. The primary distinction between the tasks resides solely in the output dimension of the classification head, which is tailored to the specific number of target classes: 6 for vocabulary classification and 24 for tonal-vowel classification.

**Table 5:** Hyperparameters and architectural configurations for EEG-conformer.

Component	Parameter	Value/configuration
Patch embedding	Temporal convolution	(1 × 25), 40 filters, stride 1
	Spatial convolution	(128 × 1), 40 filters, stride 1
	Activation and norm	ELU, batch normalization
	Average pooling	(1 × 30), stride (1 × 6)
	Embedding dropout	*p* = 0.5
Transformer encoder	Number of blocks	3
	Attention heads	10
	Embedding dimension	40
	Feed-forward expansion	4 (160 units)
	Encoder dropout	*p* = 0.5
Classification head	Feature aggregation	Global average pooling (1D)
	Fully connected layers	40 → 256 → 64 → *n*_*classes*
	Activation and dropout	ELU, *p* = {0.5, 0.3}
Optimization	Optimizer	Adam (β_1_ = 0.5, β_2_ = 0.999)
	Learning rate	2 × 10^−4^
	Batch size	16
	Training epochs	500

#### 3.2.3 Multi-task learning architecture

To mine the intrinsic correlations between neural representations that reside at the vocabulary level and those at the tonal-vowel level, a multi-task learning framework is developed based on the EEG-Conformer. This architecture aims to extract generalized spatiotemporal features that remain robust across different linguistic granularities while maintaining task-specific discriminative power. The multi-task learning model adopts a hierarchical parameter-sharing strategy, which provides a robust regularization effect to mitigate overfitting while minimizing the overall parameter count. Unlike standard hard-sharing models, this architecture supports a configurable bifurcation point within the encoder. Specifically, while the initial patch embedding module is shared, the subsequent D = 3 Transformer encoder blocks can be either shared or allocated to independent task-specific branches to optimize feature extraction for the vocabulary and tonal-vowel tasks. By forcing the model to simultaneously minimize losses from both task domains, the shared backbone is incentivized to ignore specific noise of trial and focus on discovering universal neural oscillation patterns derived from tonal-vowel and vocabulary.

To investigate the optimal layer depth at which the model should transition from shared to task-specific feature extraction, four distinct branching strategies are established: (i) Global Sharing, where the entire Encoder backbone is shared; (ii) Deep-Layer (2/3), where the first two Transformer blocks are shared; (iii) Mid-Layer (1/2), which bifurcates after the first Transformer block and the attention sub-layer of the second block; (iv) Early-Layer (1/3), where branching begins immediately after the first block. Depending on the selected branching strategy, the architecture bifurcates into two task-specific streams, each processing features through its remaining independent Transformer blocks before reaching the classification heads. Each classification head consists of a global average pooling layer and a multi-layer perceptron (MLP). The vocabulary head is optimized for a 6-class classification task, whereas the tonal-vowel head handles a more complex 24-class task. Furthermore, due to the discrepancy in neural oscillation patterns evoked by tonal-vowel and vocabulary tasks as well as the varying loss decay rates caused by classification difficulty, a dynamic loss weighting mechanism based on homoscedastic uncertainty is utilized to balance the different convergence speeds (Kendall et al., 2018). The total multi-task L_*total*_ is defined as:


$$\begin{array}{l} L_{total}=\underset{t\in \left\{v,p\right\}}{\sum }\left(\exp\left(-\log\sigma_{t}^{2}\right)L_{t}+\log\sigma_{t}^{2}\right) \end{array}$$ (2)

where L_*v*_ and L_*p*_ represent the cross-entropy losses of the vocabulary task and the tonal-vowel task, respectively. The terms $\log\sigma_{v}^{2}$ and $\log\sigma_{p}^{2}$ are learnable parameters representing task-dependent observation noise. This mechanism allows the model to automatically adjust weights during the early and late stages of training. For example, if the vocabulary task converges, the model automatically decreases its weight and shifts focus to the tonal-vowel task that has not yet converged. The training is conducted using the Adam optimizer with a joint learning rate of 0.0002, and specific experimental parameters are detailed in Table 6.

**Table 6:** Hyperparameters and architectural configurations for multi-task EEG-conformer.

Component	Parameter	Value/configuration
Encoder backbone	Embedding dimension	40
	Transformer blocks	3
	Attention heads	10
Vocabulary head	Output classes	6
	Hidden layers	40 → 128 → 64 → 6
	Dropout strategy	{0.5, 0.3}
Tonal-vowel head	Output classes	24
	Hidden layers	40 → 256 → 128 → 24
	Dropout strategy	{0.5, 0.3}
Condition head	Output classes	4
	Hidden layers	40 → 256 → 128 → 4
	Dropout strategy	{0.5, 0.3}
Loss optimization	Weighting strategy	Uncertainty-based
	Learnable parameters	$\log\sigma_{v}^{2},\log\sigma_{p}^{2}$
	Initial log-variance	0
Training settings	Total epochs	300
	Learning rate	2 × 10^−4^

In addition to linguistic decoding, the multi-task framework incorporates an experimental condition classification task. This task utilizes datasets of tonal-vowel or vocabulary collected under 4 distinct conditions to evaluate the capability of the shared backbone network to extract discriminative features across various simulated conditions, including *Overt, Overt-noisy, Intend*, and *Imagine*. The model is designed to capture different neural oscillations induced by these experimental conditions, thereby justifying the construction of the multi-condition dataset through the resulting classification performance. The specific hyperparameters for the condition classification task are presented in the Condition Head section of Table 6.

## 4 Results

### 4.1 Performance evaluation of STFT-SVM

To evaluate the decodability of the Mandarin multi-condition EEG dataset, we assess the performance of the STFT-SVM framework. Table 7 presents the classification metrics for the eight datasets. Specifically, Acc-T(%) and Acc-V(%) denote the individual classification accuracies for Mandarin tones and vowels, respectively. These metrics are decomposed from the integrated 24-class tonal-vowel classification task. Additionally, the table incorporates the Chi-square (χ^2^) statistic, the corresponding *p*, and Cramér's *V* coefficient to provide a statistical assessment of the classification results. While the χ^2^ and *p* are utilized to determine whether the model's performance significantly exceeds the theoretical chance level, Cramér's *V* is employed to quantify the effect size, thereby characterizing the strength of the association between the predicted categories and the actual labels. For the Chi-square tests, the degrees of freedom is set to 1, as the classification outcomes are binary-categorized into “correct” and “incorrect” to evaluate the deviation from the theoretical chance distribution. In the 6-class vocabulary task, the STFT-SVM achieves a peak accuracy of 38.96% under the overt condition, which significantly exceeds the theoretical chance level of 16.67% and achieves classification accuracies of 25.62% and 18.75% in the challenging inner speech decoding tasks under the intend and imagine conditions. For the 24-class tonal-vowel task, although the overall accuracy is relatively modest due to the complexity of the 24 categories, the results consistently remain above the chance level. Based on the results of the tonal-vowel classification task, we derive the individual classification performance for vowels and tones. The six-class vowel classification task reaches a maximum accuracy of 31.25%, and the four-class tone classification task attains a peak accuracy of 34.31%. Notably, tone classification accuracy generally exceeds that of vowel. The Chi-square tests proved that the STFT-SVM model successfully extracts language-related EEG features across most datasets and enables reliable classification.

**Table 7:** Classification results of STFT-SVM.

Task type	Acc(%)	Prec(%)	Recall(%)	F1(%)	Acc-T(%)	Acc-V(%)	χ^2^	*p*	*V*
Tonal-Vowel-Overt	11.53	12.27	11.53	11.46	34.31	31.25	19.54	< 0.001	0.368
Tonal-Vowel-Overt-noisy	7.22	7.75	7.22	6.96	26.81	23.75	3.36	0.067	0.153
Tonal-Vowel-Intend	6.81	7.05	6.81	6.38	24.86	25.00	2.52	0.112	0.132
Tonal-Vowel-Imagine	5.00	4.64	5.00	4.59	25.42	17.36	0.25	0.617	0.042
Vocabulary-Overt	38.96	40.66	38.96	39.04	-	-	34.34	< 0.001	0.598
Vocabulary-Overt-noisy	31.67	32.12	31.67	30.95	-	-	15.55	< 0.001	0.403
Vocabulary-Intend	25.62	25.67	25.62	25.04	-	-	5.53	0.019	0.240
Vocabulary-Imagine	18.75	18.17	18.75	18.06	-	-	0.30	0.584	0.056

### 4.2 Performance evaluation of EEG-conformer

Building upon the baseline established by the STFT-SVM framework, we evaluate the performance of EEG-Conformer. Table 8 presents the detailed classification metrics across the eight datasets, utilizing the same statistical definitions and parameters as described for Table 7. As indicated by these results, the deep learning approach yields a substantial performance gain in every experimental condition. In the 6-class vocabulary task, EEG-Conformer achieves a peak accuracy of 69.83% during overt speech, which nearly doubles the decoding capability of the linear STFT-SVM. This performance trend extends to the intend condition, where the model maintains a high accuracy of 61.46%. The accuracies of the 24-class tonal-vowel task range from 26.39% in the overt condition to 9.03% in the imagine condition, both of which consistently remain significantly above the theoretical chance level of 4.17%. Chi-square statistics confirm the statistical validity of these findings, with *p* staying below 0.001 for most conditions.

**Table 8:** Classification results of EEG-conformer.

Task type	Acc(%)	Prec(%)	Recall(%)	F1(%)	Acc-T(%)	Acc-V(%)	χ^2^	*p*	*V*
Tonal-Vowel-Overt	26.39	26.24	26.39	25.3	45.83	50	178.09	< 0.001	1.112
Tonal-Vowel-Overt-noisy	21.53	23.31	21.53	20.57	37.5	39.58	108.70	< 0.001	0.869
Tonal-Vowel-Intend	11.11	7.25	11.11	7.1	33.33	23.61	17.39	< 0.001	0.347
Tonal-Vowel-Imagine	9.03	9.48	9.03	9.1	29.17	20.14	8.53	0.003	0.243
Vocabulary-Overt	69.83	70.4	69.83	68.87	-	-	195.08	< 0.001	1.425
Vocabulary-Overt-noisy	57.29	58.23	57.29	57.12	-	-	114.08	< 0.001	1.090
Vocabulary-Intend	61.46	61.83	61.46	61.23	-	-	138.68	< 0.001	1.202
Vocabulary-Imagine	32.29	32.56	32.29	32.19	-	-	16.88	< 0.001	0.419

### 4.3 Multi-task learning results

The multi-task learning framework was evaluated under two distinct classification paradigms: (i) the joint decoding of vocabulary and tonal-vowel features within each specific experimental condition, (ii) the classification of the four experimental conditions within a fixed linguistic granularity.

Detailed classification accuracies for vocabulary, tonal-vowel, and their average performance are presented in Table 9. For the Overt and Overt-noisy conditions, the Deep-Layer (2/3) strategy consistently achieved the highest accuracy. In the Overt condition, vocabulary classification peaked at 69.79%, while the average accuracy reached 45.66%. A similar trend was observed in the Overt-noisy condition, where the Deep-Layer (2/3) strategy yielded a average accuracy of 39.59%, outperforming the Global Sharing approach by a margin of 3.99%. As the tasks transitioned toward inner speech, the optimal branching point shifted to earlier stages of the encoder. In the Intend condition, the Mid-Layer (1/2) strategy demonstrated the best performance, with vocabulary accuracy reaching 63.54% and average accuracy at 36.29%. Conversely, for the Imagine condition, which represents the highest level of cognitive abstraction, the Early-Layer (1/3) strategy attained the highest results, with vocabulary and average accuracies of 32.29% and 18.38%, respectively. Notably, the vocabulary classification results under the Overt-noisy and Intend conditions were better than those obtained with the single-task EEG-Conformer model. The overall classification performance is consistent with the patterns observed in single-task experiments. As the experimental task transitions from overt speech to inner speech, the overall accuracy exhibits a downward trend. Regarding the condition classification task, the multi-task framework demonstrated high discriminative power in distinguishing between the four experimental states (Overt, Overt-noisy, Intend, and Imagine). As detailed in Table 10, the average accuracy for condition classification exceeded 97% for both the tonal-vowel and vocabulary data.

**Table 9:** Classification accuracy of different multi-task architecture configurations across four EEG conditions.

Condition	Branching strategy	Vocabulary (%)	Tonal-vowel (%)	Average (%)
Overt	Global Sharing	57.29	17.36	37.33
	Deep-Layer (2/3)	**69.79**	21.53	**45.66**
	Mid-Layer (1/2)	58.33	18.06	38.20
	Early-Layer (1/3)	54.17	**22.22**	38.20
Overt-noisy	Global Sharing	59.38	11.81	35.60
	Deep-Layer (2/3)	**60.42**	**18.75**	**39.59**
	Mid-Layer (1/2)	58.33	**18.75**	38.54
	Early-Layer (1/3)	57.29	13.19	35.24
Intend	Global Sharing	53.12	4.86	28.99
	Deep-Layer (2/3)	53.12	6.94	30.03
	Mid-Layer (1/2)	**63.54**	9.03	**36.29**
	Early-Layer (1/3)	56.25	**9.72**	32.99
Imagine	Global Sharing	26.04	**6.94**	16.49
	Deep-Layer (2/3)	26.04	5.56	15.80
	Mid-Layer (1/2)	27.08	6.25	16.67
	Early-Layer (1/3)	**32.29**	4.47	**18.38**

The highest accuracy in each condition is highlighted in bold.

**Table 10:** Classification accuracy of experimental conditions based on different linguistic granularities.

Data Granularity	Overt (%)	Overt-noisy (%)	Intend (%)	Imagine (%)	Average (%)
Tonal-vowel	99.34	98.21	99.56	97.30	98.60
Vocabulary	99.84	99.72	99.22	98.73	99.38

### 4.4 Electrode ranking and ablation results

**Figure 4:**
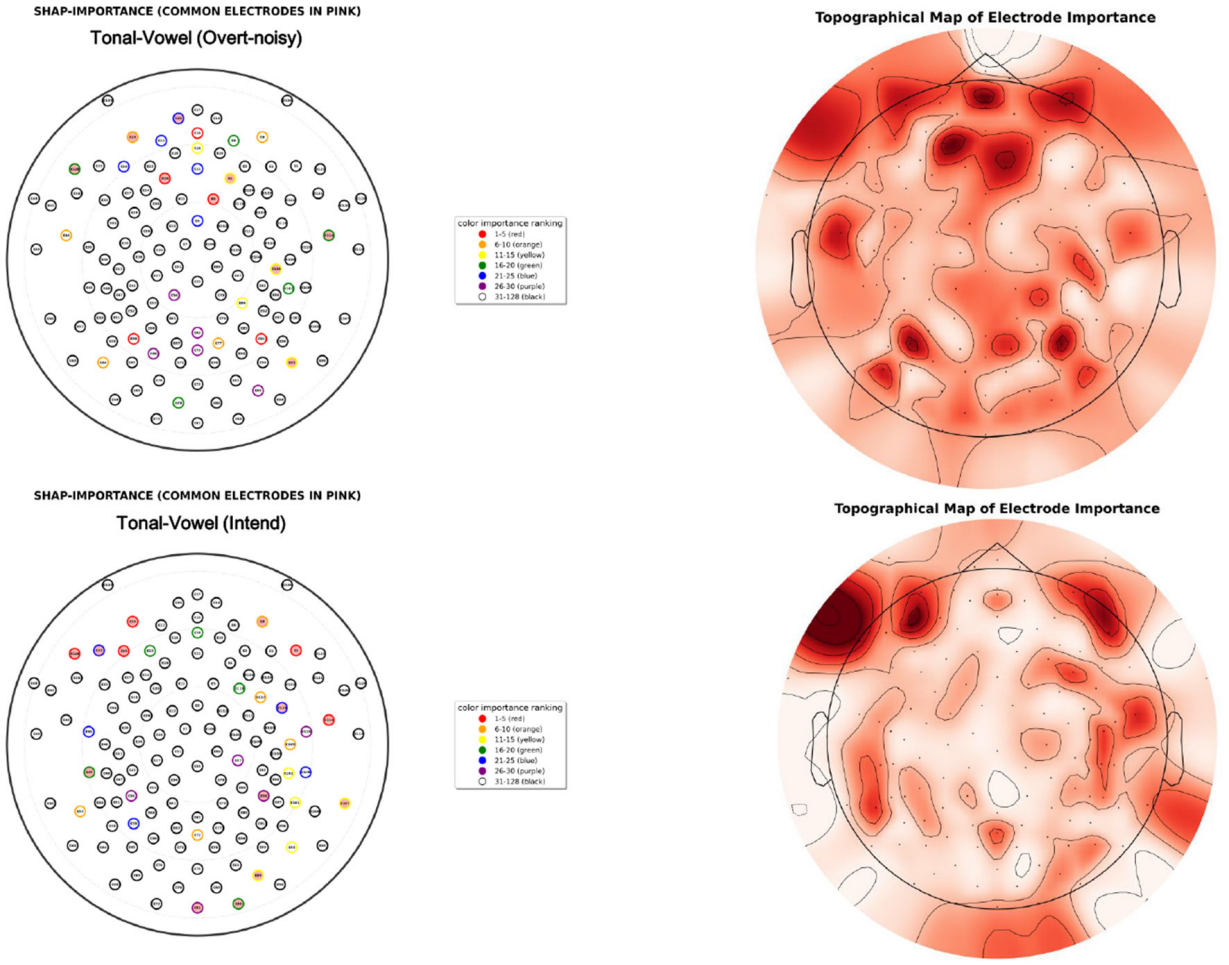
The figure illustrates the electrode importance analysis for the Tonal-Vowel paradigm under overt-noisy and intend conditions. (**Left**) Discrete electrode importance rankings, where the electrodes shaded in pink indicate the intersection of the top 30 electrodes identified by both SHAP values and RF feature importance. (**Right**) The corresponding topographical maps based on spatial interpolation, illustrating the continuous spatial distribution of electrode importance across the scalp.

**Figure 5:**
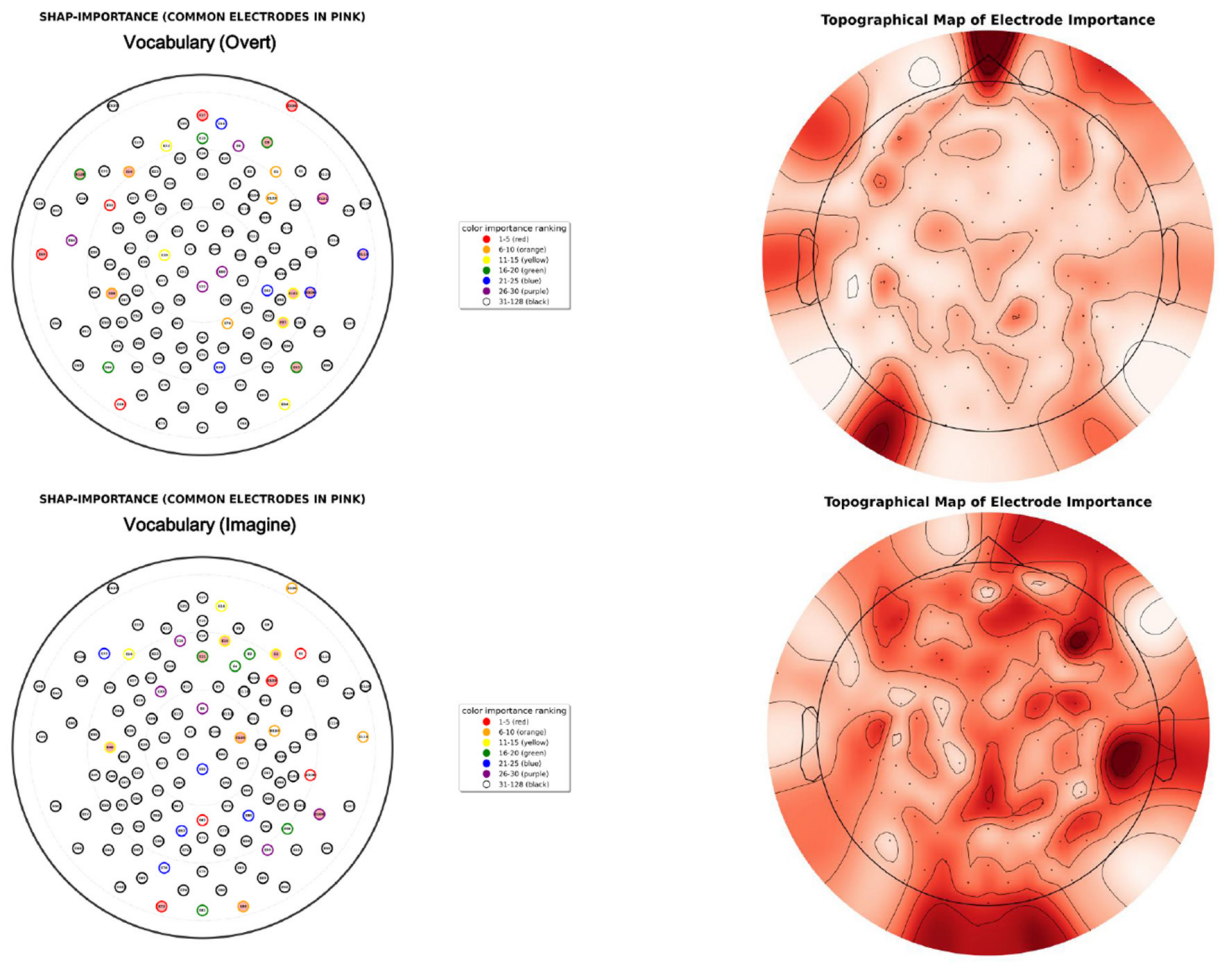
The figure illustrates the electrode importance analysis for the Vocabulary paradigm under overt and imagine conditions. (**Left**) Discrete electrode importance rankings, where the electrodes shaded in pink indicate the intersection of the top 30 electrodes identified by both SHAP values and RF feature importance. (**Right**) The corresponding topographical maps based on spatial interpolation, illustrating the continuous spatial distribution of electrode importance across the scalp.

To evaluate the contribution of individual channels, we calculate the importance of different electrodes in the STFT-SVM task using two distinct methods. Specifically, we compute the SHAP values for the STFT-extracted window features to determine their impact on classification outcomes, alongside employing the inherent feature importance metric of a RF classifier. This approach yields two independent electrode importance rankings for each classification experiment. Figures 4, 5 illustrates the detailed results of a portion of the dataset, where the electrodes shaded in pink indicate the intersection of the top 30 electrodes identified by both approaches. We compute the union of the top 30 electrodes identified by SHAP and RF importance rankings. Utilizing this specific electrode subset as the input for both the STFT-SVM and EEG-Conformer models. Tables 11, 12 present the results, in which Channels denotes the number of electrodes utilized, and Acc improvement represents the gain in accuracy relative to the baseline performance achieved by the use of all electrodes. Following the optimization of the number of electrodes, the classification performance of the model remained comparable to the level attained when the complete set of electrodes was employed.

**Table 11:** Classification results of selected electrodes for the STFT-SVM.

Task type	Acc(%)	Prec(%)	Recall(%)	F1(%)	Channels	Acc improvement(%)
Tonal-Vowel-Overt	16.39	15.02	16.39	15.23	46	+4.86
Tonal-Vowel-Overt-noisy	6.67	6.12	6.67	6.31	51	-0.55
Tonal-Vowel-Intend	6.85	6.77	6.85	6.69	46	+0.04
Tonal-Vowel-Imagine	4.72	4.49	4.72	4.53	47	+0.28
Vocabulary-Overt	40.62	41.02	40.62	40.67	43	+1.66
Vocabulary-Overt-noisy	32.92	32.94	32.92	32.74	47	+1.25
Vocabulary-Intend	29.58	29.16	29.58	29.22	45	+3.96
Vocabulary-Imagine	19.58	20.17	19.58	19.77	51	+0.83

**Table 12:** Classification results of selected electrodes for the EEG-conformer.

Task type	Acc(%)	Prec(%)	Recall(%)	F1(%)	Channels	Acc improvement(%)
Tonal-Vowel-Overt	29.86	31.25	29.86	29.15	46	+3.47
Tonal-Vowel-Overt-noisy	26.39	27	26.39	25.85	51	+4.86
Tonal-Vowel-Intend	12.5	9.53	12.5	10.36	46	+1.39
Tonal-Vowel-Imagine	7.64	7.07	7.64	6.94	47	-1.39
Vocabulary-Overt	69.79	69.95	69.79	69.79	43	+0.04
Vocabulary-Overt-noisy	67.71	68.14	67.71	67.88	47	+10.42
Vocabulary-Intend	72.92	75.18	72.92	73.02	45	+11.46
Vocabulary-Imagine	25	23.99	25	23.71	51	-7.29

## 5 Discussion

This study constructs a multi-condition Chinese EEG dataset simulating various conditions of speech impairment and validates an integrated framework for speech decoding. Our evaluation confirms the high distinguishability of the dataset. We benchmarked mainstream architectures for speech decoding and designed a joint multi-task learning architecture for tonal-vowel and vocabulary. Furthermore, an interpretability approach based on SHAP and RF enabled us to reduce the required electrodes by more than half while maintaining high classification accuracy.

### 5.1 Performance analysis of different models for speech decoding

Regarding research question (1), Both the STFT-SVM and EEG-Conformer frameworks achieve classification accuracies that significantly surpass chance levels across all evaluated tasks. The EEG-Conformer architecture consistently demonstrates superior performance compared to STFT-SVM, which is a traditional machine learning method. This advantage is particularly pronounced in the vocabulary classification task under the intend condition, which is a condition designed to simulate speech communication impairments. In this case, the accuracy of EEG-Conformer reaches 69.83%, whereas the accuracy of STFT-SVM is limited to 31.67%. In comparison with classification tasks on foreign language datasets, Brigham and Kumar (2010) collect and classify imagined speech data from seven subjects. They perform a binary classification task on foreign syllables ba and ku, achieving a classification rate of 61% against a chance level of 50%. Relative to the chance level, our model demonstrates comparable performance. In a related study, van den Berg et al. (2021) apply the EEGNet model to an inner speech classification task that evaluates eight subjects on four simple foreign vocabularies including up, down, left, and right. They report an average accuracy of 29.67%, while our inner speech experiments yield accuracies of 61.46% and 32.29%. These comparative results indicate that the proposed research successfully verifies the decodability of the multi-condition Mandarin EEG dataset utilizing the EEG-Conformer framework. The model accomplishes reliable classification particularly under the two conditions that simulate individuals with speech communication impairments. Such results suggest that the self-attention mechanism within the architecture effectively extracts subtle neural signatures of speech intention that traditional features fail to isolate (Abibullaev et al., 2023; Song et al., 2022).

We observe a systematic performance gradient where accuracy declines substantially as the paradigm shifts from overt speech to inner speech, such as intend and imagine task, with the most pronounced decrease occurring in the imagine condition. The systematic performance gradient provides insights into the neurophysiological differences between overt and inner speech. As accuracy declines substantially from overt to imagine conditions, we infer that this is due to the varying complexity of signal processing layers. Hickok and Poeppel (2007) noted that overt speech involves the simultaneous processing of three signal layers: motor commands (electrical signals sent to the vocal organs), auditory feedback (hearing one's own voice), and somatosensory feedback (tactile sensations from the contact of the tongue and lips). Unlike overt speech, which benefits from robust external feedback, imagine and intend tasks primarily involve motor commands without direct acoustic output. Consequently, the absence of these sensory feedback mechanisms directly leads to the lower classification accuracies observed in the intend and imagine tasks.

Furthermore, drawing on the theoretical framework of György Buzsáki (2019), which highlights how the brain anchors internal representations to external environmental cues, we infer that the performance degradation in inner speech stems from a lack of external temporal anchoring. While overt speech is stabilized by the rhythms of the physical world, resulting in consistent speech rates and motor actions, the internal pacing of inner tasks is inconsistent. This lack of external constraints introduces substantial temporal variance even within the same subject across different trials. Consequently, the neural oscillations generated by inner speech tasks exhibit lower signal-to-noise ratios, higher temporal variability, and more dispersed representations, posing significant challenges for high-precision decoding using traditional feature extraction and linear classification methods.

### 5.2 Analysis of multi-task learning mechanisms for speech decoding

To address research questions (1) and (2), we develop a multi-task architecture to investigate whether EEG data from distinct tasks under identical experimental conditions can mutually enhance representation learning, and whether significant differences exist across the datasets. This approach draws inspiration from the encoder designs of Large Brain Model (Jiang et al., 2024), which utilize joint training across diverse datasets to mitigate the limitations of specific models and extract generalized EEG features. Specifically, we hypothesize that a shared multi-task encoder can simultaneously learn the common neurophysiological patterns underlying both tonal-vowel and vocabulary processing, thereby facilitating the generation of more robust feature representations for Mandarin EEG signals. However, contrary to this expectation, the classification performance for both vocabulary and tonal-vowel within the multi-task framework exhibits a decline when compared to the independent decoding results obtained from the single-task EEG-Conformer baseline.

The observed decline in performance within the multi-task framework can be explained by the phenomenon of negative transfer (Pan and Yang, 2009). This stems from significant imbalances in data scale and category complexity between the 6-class vocabulary task and the 24-class tonal-vowel task. Moreover, the result reflects a fundamental difference in cognitive processing: vocabulary processing involves high-level semantic comprehension, whereas tonal-vowel processing relies more on low-level cortical responses. The attempt to find a shared feature manifold for both high-level semantics and low-level phonemes consequently weakens the discriminative capability of each individual task. Furthermore, experimental results demonstrate that the hierarchical parameter-sharing strategy within the multi-task framework yields substantial improvements in classification accuracy relative to the global sharing paradigm. By optimizing the locations of task-specific branching points, negative transfer in multi-task learning is effectively mitigated. A notable trend is observed: as the speech condition transitions from overt speech toward inner speech, the optimal branching point within the Transformer modules progressively shifts to earlier stages. In the Overt and Overt-noisy conditions, the superior performance of the Deep-Layer (2/3) strategy suggests that during the execution of actual speech, both tasks share extensive low-level motor-sensory pathways and phonatory commands within the motor cortex. Such shared spatiotemporal features facilitate deeper parameter coupling without mutual interference. Conversely, in the Intend and Imagine conditions, the Mid-Layer (1/2) and Early-Layer (1/3) strategies achieve the highest performance. This indicates that in the absence of overt motor movement, forced deep sharing across these abstract cognitive states results in feature dilution, whereas earlier branching enables the model to preserve task-specific nuances of internal mental imagery more effectively. Conversely, the exceptional accuracy (exceeding 97%) in the condition classification task confirms the validity of our multi-condition dataset. This indicates that different articulatory or cognitive states elicit distinct and prominent neural oscillation patterns in the cerebral cortex, effectively simulating the clinical states of patients with varying speech communication impairments.

### 5.3 Interpretability-driven channel reduction and its practical implications

Regarding the content of research question (3). Experimental observations demonstrate that the rankings generated by SHAP values for the STFT-extracted window features and the inherent feature importance metric of a RF classifier exhibit a high degree of overlap, thereby improving the reliability of the identified significant channels. This model interpretability method enables the quantitative assessment of contributions from distinct channels and features to the resulting classification decisions. EEG speech decoding is characterized by significant complexity. Traditional research typically favors high-density systems, such as 128-channel configurations, because they provide comprehensive brain coverage. This spatial resolution facilitates the capture of subtle neural features, which generally yields higher classification accuracy than sparse-electrode setups. Building upon these findings, we conduct an ablation study where the union of the top 30 electrodes identified by SHAP and RF importance rankings serves as the new feature input for the STFT-SVM model. Despite a channel reduction of over 50%, the classification performance improves across almost all evaluated tasks.

The future development of the BCI field involves mass production and clinical deployment. High-density devices face practical challenges in real-world applications, such as the need for continuous replenishment of conductive moisture to maintain electrode impedance and the high associated hardware costs. Therefore, the design of portable BCIs must prioritize lightweight and mobile architectures, necessitating a significant reduction in the number of electrodes. Our experimental results offer a theoretical basis for this direction. For speech decoding tasks involving individuals with speech communication disorders in China, future BCI systems can selectively place electrodes over specific critical brain regions. This approach reduces hardware costs and usability barriers while allowing the system to focus on capturing task-relevant neural oscillation patterns.

### 5.4 limitation and future work

Despite the promising results, this study has several limitations that point toward meaningful directions for future research. First, the current dataset is constructed with a relatively limited number of subjects, and the evaluations primarily focus on within-subject decoding. Given the inherent non-stationarity and high inter-subject variability of EEG signals, practical BCI systems must account for cross-subject generalization. Future efforts will focus on expanding the cohort size and employing advanced transfer learning or domain adaptation techniques to develop robust, subject-independent decoding models.

Second, although the proposed multi-task learning architecture provides an integrated framework, its performance in the joint decoding of tonal-vowel and vocabulary is suboptimal, revealing a noticeable negative transfer phenomenon. This interference between different linguistic granularities suggests that shared feature representations may struggle to simultaneously balance fine-grained phonetic traits and coarse-grained semantic information. To address this, future research will explore more sophisticated network designs or increase the volume of training data to mitigate this negative transfer.

Finally, natural human communication relies on continuous speech. Therefore, expanding the data collection paradigm to construct continuous, sentence-level Chinese Mandarin EEG datasets will be a critical next step. Furthermore, we aim to collect real-world data from individuals with speech communication disorders.

## 6 Conclusion

This study systematically constructs a multi-condition Chinese EEG dataset simulating various conditions of speech impairment and validates an integrated framework for speech decoding. By incorporating four distinct communication conditions, the dataset demonstrates exceptional neural distinguishability and achieves condition classification accuracies exceeding 97%. Comprehensive evaluations utilizing both traditional machine learning and deep neural networks confirm the strong decodability of the collected data. Additionally, joint multi-task learning experiments reveal negative transfer phenomena between different linguistic granularities in tonal-vowel and discrete vocabulary. Furthermore, an interpretability approach utilizing SHAP and RF about channel contributions, which facilitates an electrode reduction of over fifty percent without compromising classification performance. This spatial optimization significantly diminishes computational overhead and hardware complexity, thereby proposing a technical foundation for portable and real time BCIs. Future research will expand upon this paradigm to address inter subject variability and encompass continuous sentence level speech decoding.

## Data Availability

The raw data supporting the conclusions of this article will be made available by the authors, without undue reservation.
